# Feliform carnivores have a distinguished constitutive innate immune response

**DOI:** 10.1242/bio.014902

**Published:** 2016-04-04

**Authors:** Sonja K. Heinrich, Bettina Wachter, Ortwin H. K. Aschenborn, Susanne Thalwitzer, Jörg Melzheimer, Heribert Hofer, Gábor Á. Czirják

**Affiliations:** 1 Leibniz Institute for Zoo and Wildlife Research, Alfred-Kowalke-Str. 17, Berlin 10315, Germany; 2 Bwabwata Ecological Institute, Ministry of Environment and Tourism, Zambezi, Namibia

**Keywords:** Bacterial killing assay, Carnivores, Constitutive innate immunity, Canids, Felids

## Abstract

Determining the immunological phenotype of endangered and threatened populations is important to identify those vulnerable to novel pathogens. Among mammals, members of the order Carnivora are particularly threatened by diseases. We therefore examined the constitutive innate immune system, the first line of protection against invading microbes, of six free-ranging carnivore species; the black-backed jackal (*Canis mesomelas*), the brown hyena (*Hyena brunnea*), the caracal (*Caracal caracal*), the cheetah (*Acinonyx jubatus*), the leopard (*Panthera pardus*) and the lion (*Panthera leo*) using a bacterial killing assay. The differences in immune responses amongst the six species were independent of their foraging behaviour, body mass or social organisation but reflected their phylogenetic relatedness. The bacterial killing capacity of black-backed jackals, a member of the suborder Caniformia, followed the pattern established for a wide variety of vertebrates. In contrast, the five representatives of the suborder Feliformia demonstrated a killing capacity at least an order of magnitude higher than any species reported previously, with a particularly high capacity in caracals and cheetahs. Our results suggest that the immunocompetence of threatened felids such as the cheetah has been underestimated and its assessment ought to consider both innate and adaptive components of the immune system.

## Abstract (precis)

**Summary:** The innate immunocompetence of six free-ranging carnivores is independent of their foraging behaviour, body mass or social organisation but reflects their phylogenetic relatedness.

## INTRODUCTION

One key factor threatening mammalian wildlife populations are pathogens and parasites (Smith et al., 2009). Members of the order Carnivora are particularly threatened by diseases; according to the IUCN red list, 25% of carnivores are considered as threatened and within those 27% of the 30 species in the family Canidae and 8% of the 36 species in the family Felidae are threatened by diseases (Pedersen et al., 2007; Smith et al., 2009). Knowing the immunocompetence of threatened species is therefore particularly important for disease management because it is a critical aspect of disease resistance and thus survival (Graham et al., 2011). In carnivores, species feeding on carrion are more likely to be infected than species feeding exclusively on freshly killed prey because of a higher abundance of pathogens colonizing carrion (DeVault et al., 2003). Larger species may also have a higher infection risk than smaller species because larger bodies need more food and may harbour more pathogens than smaller ones (Kuris et al., 1980; Morand and Poulin, 1998; Schneeberger et al., 2013; but see Vitone et al., 2004). Social species live under higher pathogen pressure than solitary ones because of higher intraspecific contact rates and closer proximity of individuals (Altizer et al., 2003). Additionally, higher rates of intraspecific horizontal transmission and multiple infections are associated with the evolution of increased virulence which is promoted by social species (Wilson et al., 2003).

Pathogen transmission risk and virulence should be reflected in species-specific patterns of immunocompetence because pathogens impose a strong selective pressure on their hosts (Schneeberger et al., 2013; Wilson et al., 2003). Thus, species with a high transmission risk should maintain a higher immunocompetence than species with a low transmission risk. Alternatively, closely related species might exhibit a similar immunocompetence because many immune system components are genetically encoded as many other traits and might be shared through common ancestors (Caroll and Prodeus, 1998; Lee, 2006; Ochsenbein and Zinkernagel, 2000).

Here we investigate the strength of the constitutive innate immune system in six free-ranging carnivore species from the same carnivore guild with different foraging behaviour, body size and social organisation (Table 1) using a bacterial killing assay (BKA). This assay is useful because it measures a functional response of the innate immune system of an animal (Tieleman et al., 2005). The BKA determines the ability to eliminate a pathogen encountered and has been demonstrated to be an excellent predictor of the susceptibility to a variety of bacterial infections in humans (Keusch et al., 1975). It is easily adaptable to different species without the need of species specific reagents, which makes it suitable for comparative studies (Millet et al., 2007; Schneeberger et al., 2013). Its interpretation is clear and straightforward and it has been successfully used to estimate constitutive innate immunity in many different species, e.g. in bats (Schneeberger et al., 2013), gazelles (Ezenwa et al., 2012), spotted hyena (Flies et al., 2015), skinks (Kuo et al., 2013) and many bird species (Matson et al., 2006; McGraw and Klasing, 2006; Millet et al., 2007). The constitutive part of the innate immune system is always present at low levels in the blood and provides a rapid, first line defences against parasites and pathogens (Janeway et al., 2001).

**Table 1.**
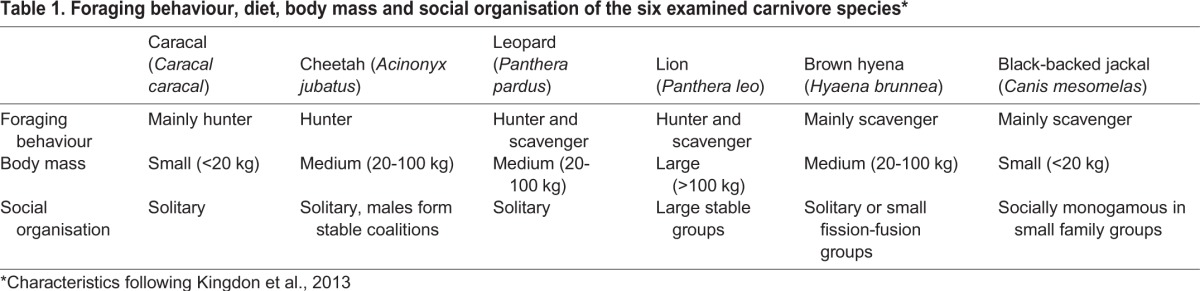
Foraging behaviour, diet, body mass and social organisation of the six examined carnivore species*

We used four felids; the caracal (*Caracal caracal*), cheetah (*Acinonyx jubatus*), leopard (*Panthera pardus*) and lion (*Panthera leo*), and the brown hyena (*Hyaena brunnea*, family Hyaenidae), all from suborder Feliformia, and one canid, the black-backed jackal (*Canis mesomelas*) from suborder Caniformia. If foraging behaviour is the best predictor for the constitutive innate immunocompetence, brown hyenas and black-backed jackals should have the highest immunocompetence and cheetahs the lowest, if body size is the best predictor, lions should have the highest immunocompetence and black-backed jackals and caracals the lowest, and if social organisation is the best predictor, lions should have the highest values and leopards and caracals the lowest. Alternatively, the immunocompetence follows the phylogenetic relationships of the species.

## RESULTS

**Fig. 1.**
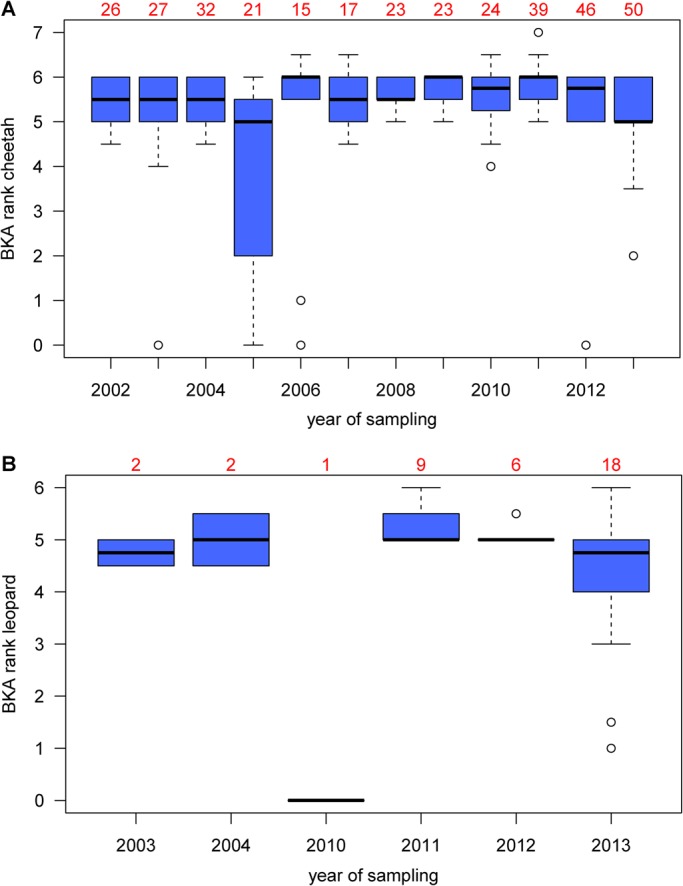
**BKA ranks of samples from different years**. For cheetahs (A) and leopards (B). Numbers above boxplots represent sample sizes. Circles depict values more than 1.5 times the interquartile range below the first quartile. Changes between years were not significant.

Samples were stored between six months and 11 years, and thawed for the first time. There was no difference in BKA results between (1) measurements of the 46 animals sampled repeatedly (Skillings Mack Statistic=4.55, 10.000 permutations, *P*=0.31), (2) cheetah samples stored for different numbers of years (Jonckheere–Terpstra test, JT=26,851, 10.000 permutations, *P*=0.48; Fig. 1A) and (3) leopard samples stored for different numbers of years (JT=203.5, 10.000 permutations, *P*=0.90; Fig. 1B). We therefore used samples from all study years and chose randomly one sample of individuals sampled repeatedly to avoid pseudo-replications in the data set. Two cheetah family groups were responsible for the drop in killing capacity of cheetah serum in 2005 (Fig. 1A). The three cubs of one mother and a group of three young males had particularly low results (ranks 0, 0, 2 and 0, 0, 0, respectively). Their immune system might have been affected by illness at the time of capture although they did not show any clinical signs.

**Fig. 2.**
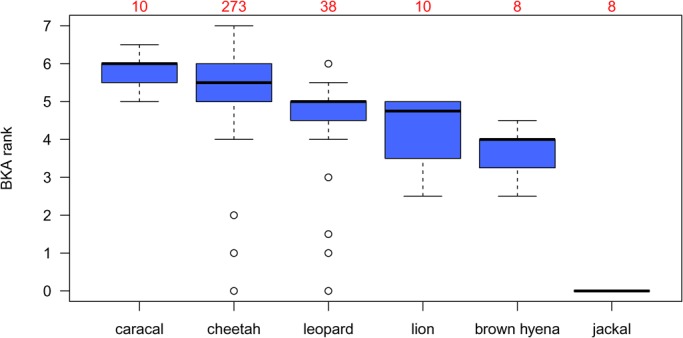
**BKA ranks of the six carnivore species.** Numbers above boxplots represent sample sizes. Circles depict values more than 1.5 times the interquartile range below the first quartile.

**Fig. 3.**
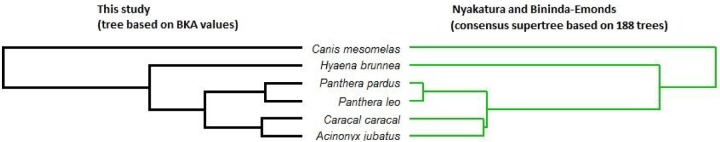
Comparison of phylogenies derived from BKA values (this study) and the supertree reported by Nyakatura and Bininda-Emonds (2012). BKA results were hierarchically clustered with the centroid method (D'haeseleer, 2005).

Species differed in their BKA results (Kruskal–Wallis test, H=85.56, d.f.=5, *P*<0.001). Posthoc pairwise comparison revealed similar BKA ranks for caracals and cheetahs, for lions and leopards and for lions and brown hyenas (Table 2). The highest bacterial killing capacity was measured in caracals and cheetahs and the lowest in black-backed jackals (Fig. 2). There was a positive linear relationship between the BKA distance matrix and the genetic distances between species (Mantel test, r=0.773, *P*=0.023; Fig. 3).

**Table 2.**
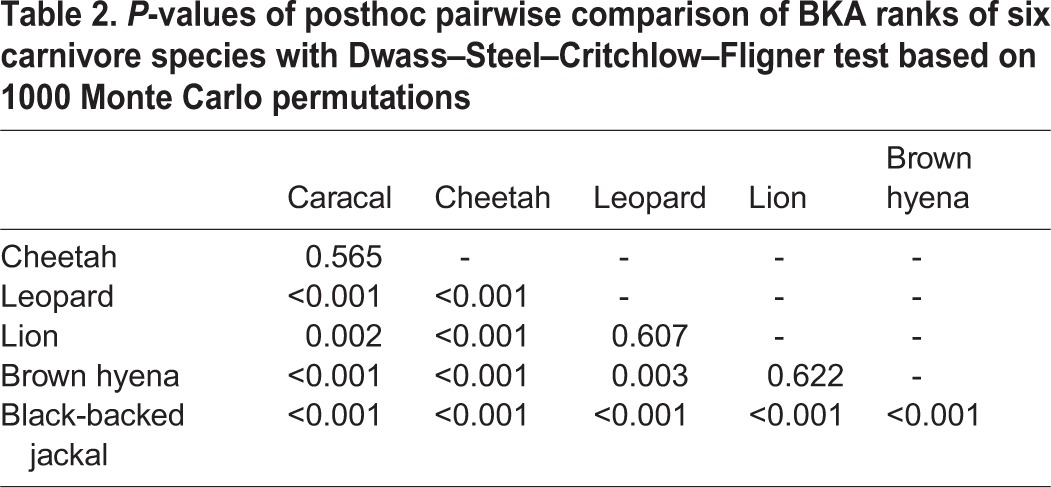
***P*-values of posthoc pairwise comparison of BKA ranks of six carnivore species with Dwass–Steel–Critchlow–Fligner test based on 1000 Monte Carlo permutations**

**Fig. 4.**
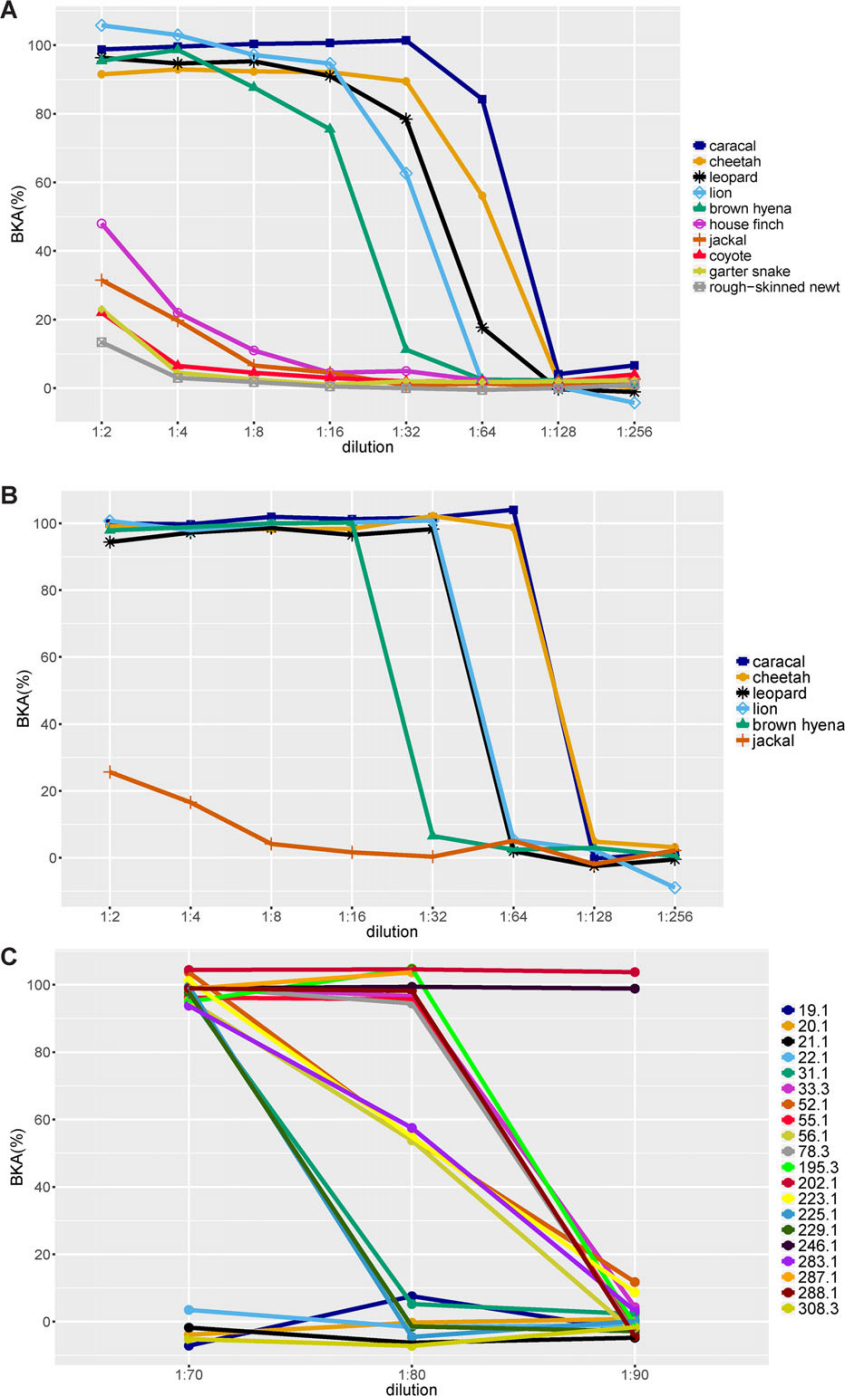
**BKA values for different species**. (A) Mean BKA values for eight serial dilutions of the six carnivore species from this study and four other species (a carnivore, a bird, a reptile and an amphibian) previously published. Values for published species are from Fig. 2A from French and Neuman-Lee (2012). (B) BKA values for eight serial dilutions of six carnivore species. Lines represent one randomly chosen individual per species. (C) BKA values for dilutions 1:70, 1:80 and 1:90 for 20 randomly selected cheetahs.

Black-backed jackals had BKA values similar to those of coyotes (*Canis latrans*), house finches [*Haemorhous* (formerly *Carpodacus*) *mexicanus*], newts (*Taricha granulosa*) or garter snakes (*Thamnophis elegans*) (French and Neuman-Lee, 2012), whereas all feliform species had substantially higher BKA values (Fig. 4A).

The complete dataset of BKA results is available in the Supplementary information.

## DISCUSSION

Within free-ranging mammals, comparative eco-immunological studies were previously available only from bats and rodents (Schneeberger et al., 2013; Tian et al., 2015), and for individual species from a variety of vertebrates (French and Neuman-Lee, 2012). Here we extend such studies by comparing the bacterial killing capacity of six sympatric carnivore species.

Storage duration had no effect on the bacterial killing capacity of carnivore serum against *Escherichia coli* (*E.coli*) when comparing samples frozen for 6 months up to 11 years. In bats, storing duration of plasma between 41 up to 81 days did not change the bacterial killing capacity (Schneeberger et al., 2013), similar to plasma of flycatchers (*Myiarchus cinerascens*) and bluebirds (*Sialia mexicana*) which retained bacterial killing capacity after 19-53 days (average 30 days) of storage at −80°C (Jacobs and Fair, 2016). Also, plasma of tree swallows (*Tachycineta bicolor*) stored for 6 months at −80°C revealed similar results as fresh plasma (Morrison et al., 2009). However, in some bird species bacterial killing capacity can also drop drastically within the first few weeks of storage. In house sparrows (*Passer domesticus*), bacterial killing capacity of plasma dropped from approximately 50% to 15% within the first three weeks when samples were stored at −40°C (Liebl and Martin, 2009), similar to the plasma of chickens (*Gallus gallus*) that dropped close to zero in most samples after 19-53 days stored at −80°C (Jacobs and Fair, 2016). These differences might be due to different storage temperatures, samples used (serum or plasma) or might reflect a difference in sensitivity of humoral effectors to storage length for different species. However, even if our samples lost bacterial killing capacity within the first few weeks, it cannot be much, because nearly all samples of the feliform carnivores had an initial bacterial killing capacity of ≥95%, irrespective of the storage time.

Foraging behaviour was not a good predictor of the innate immunocompetence of the six carnivore species. Brown hyenas and black-backed jackals, the main carrion feeders, showed the lowest BKA values instead of the predicted highest. Also, BKA values of large species were not higher than those of smaller species, because the two smallest species, caracal and black-backed jackal, showed the highest and lowest BKA values, respectively. Nor did social organization explain BKA values because lions, the most social species, showed an intermediate BKA result, whereas caracals and leopards, both solitary, had higher and similar values compared to lions, respectively.

The latter might be surprising because among mammals and other vertebrates social group size appears to be an important predictor of parasite risk (Côté and Poulin, 1995). The different branches of the immune system convey different costs and benefits and it is possible that different branches may compensate each other (Norris and Evans, 2000). Unique to adaptive immunity is the immunological memory that confers long-lasting immunity against pathogens and is advantageous in case of repeated infections with the same pathogen because its response is quick and specific (Janeway et al., 2001). Strong adaptive immunity may thus be more advantageous for social species, because social species often experience higher and repeated pathogen pressure from the same pathogen (Côté and Poulin, 1995). Consistent with this, T- and B-cell mediated adaptive immune responses were significantly stronger in colonial than non-colonial bird species (Møller et al., 2001). However, the bacterial killing assay used in this study estimates the strength of the constitutive innate immune response. Thus, sociality of a species might not influence the innate immune response but rather the adaptive immune response. Furthermore, immunity against parasites that are classified as generalists, i.e. infect many host species, and are transmitted by vectors or through contaminated soil, food or water might be less influenced by social organisation than immunity against parasites that are classified as specialists (Altizer et al., 2003). Because our BKA estimates the immune response against *E.coli*, an ubiquitous pathogen that infects many host species, sociality might not be very important for the pathogen transmission risk of this bacterium.

Of the six carnivore species we examined in this study, the cheetah is known to have a low genetic variability, including at the major histocompatibility complex (MHC), a multigene family crucial to the adaptive immune defense of vertebrates (Castro-Prieto et al., 2011b; O'Brien et al., 1983). It was suggested that the cheetah was highly susceptible to infectious diseases, but this was not confirmed for free-ranging cheetahs (Thalwitzer et al., 2010). This suggests that the different immune branches (adaptive and innate) can compensate each other and would explain the high BKA results in cheetahs. In line with this are the lower BKA results of leopards which have a higher MHC variability than cheetahs (Castro-Prieto et al., 2011a). Within the Hyaenidae, striped hyenas (*Hyaena hyaena*) and spotted hyenas (*Crocuta crocuta*) have a similar MHC variability and a higher one than cheetahs and leopards (Califf et al., 2013), but the MHC variability of brown hyenas is not described, nor the ones of caracals, African lions, and black-backed jackals. Thus, adaptive and innate immunity may compensate each other but more information for the carnivore species in this study is needed to confirm these first results. Although the bacterial killing capacity of free-ranging and captive spotted hyenas was studied, methodological differences make it difficult to compare the results of the study (Flies et al., 2015) to the ones obtained by us. Additional information on other aspects of the immune system for these species is sparse. Studies on African lions have mostly focused on infectious diseases such as tuberculosis (Viljoen et al., 2015) and canine distemper virus (Roelke-Parker et al., 1996), but a functional characterisation of their immune system has not been attempted.

The only good predictor of the strength of the constitutive innate immune system of our species was the phylogeny, because the tree based on BKA results was similar to the one representing evolutionary relationships between the species (Nyakatura and Bininda-Emonds, 2012). These results are intriguing from at least two perspectives: (1) The strong selection pressure assumed to be exerted by different environments as represented by variation in foraging behaviour, body size or social organisation did not fine-tune the innate immune system, being – in comparison with the adaptive response – the evolutionarily older part of the immune system (Janeway et al., 2001). (2) The ancestors of feliform carnivores developed a superior form of constitutive innate immune response.

All feliform species had BKA values an order of magnitude higher than those previously reported from various vertebrates and those of black-backed jackals. It is therefore unlikely that the 8% of felids threatened by diseases suffer from a weakness of their constitutive immune system.

## MATERIAL AND METHODS

### Sample collection

Between 2002 and 2013 we blood-sampled 275 cheetahs (194 males, 81 females), 38 leopards (19 males, 19 females), ten lions (7 males, 3 females), ten caracals (5 males, 4 females, 1 unknown), eight black-backed jackals (6 males, 1 female, 1 unknown) and eight brown hyenas (4 males, 4 females). Animals were free-ranging on commercial farmland in east-central Namibia, except for six lions which were sampled in Etosha National Park in north-central Namibia and in Caprivi region in north-eastern Namibia. Forty-eight cheetahs, four lions, one leopard and one caracal were wild born animals kept in large enclosures on privately owned farms or at the AfriCat Foundation, a non-profit conservation facility for carnivores in central Namibia. All free-ranging animals, except lions, were captured in box traps, immobilized as previously described (Thalwitzer et al., 2010; Wachter et al., 2011) and released again at the site of capture. Free-ranging lions were darted from a vehicle on bait or on a kill. Captive animals were immobilized in their enclosures. Thirty, eight, four, two and one cheetahs were sampled twice, three, four, five and six times, respectively, and one caracal was sampled twice, resulting in 421 samples.

Blood was taken from the cephalic vein with sterile Vacutainer^®^ serum tubes (Becton Dickinson), transported to the field laboratory in a cooler box and centrifuged within 24 h after sampling. Serum samples were sub-sampled, frozen and stored in liquid nitrogen until transported to Germany on dry ice or in liquid nitrogen, in full compliance with the Convention on International Trade and in Endangered Species (CITES), and then stored at −80°C.

The sex ratio of the species was similar (Fisher's exact test, *P*=0.090), thus we combined all samples for analyses. Animal immobilizations and sample collections were authorized by the Ministry of Environment and Tourism of Namibia and complied with the laws of the country.

### Bacterial killing assay (BKA)

To measure the constitutive immune system, we used the bacterial killing assay (BKA) (Liebl and Martin, 2009; Tieleman et al., 2005), which measures the capacity of plasma to kill microorganisms and integrates many important humoral components of constitutive innate immunity. The bacterial killing assay was conducted with *E.coli* (ATCC No 8739), a ubiquitous gram-negative bacteria, with which all studied species regularly come into contact. We followed the method proposed by French and Neumann-Lee but used larger volumes (French and Neuman-Lee, 2012). Lyophilized pellets of *E.coli* were reconstituted in 10 ml sterile phosphate-buffered saline (PBS). The reconstituted bacteria were plated on a blood-agar plate and incubated at 37°C for 24 h. One colony was transferred into 5 ml of Tryptic Soy Broth (TSB) (Sigma Aldrich, Steinheim, Germany) 4-6 h before the assay and incubated at 37°C while placed on a shaker at 170 rpm (Stuart orbital incubator S1500, Bibby Scientific, Staffordshire, UK). Directly prior to the assay, we diluted the bacteria to a concentration of McFarland 0.5. This solution was then diluted by 10^3^ with sterile PBS to obtain the bacterial working solution with an approximate concentration of 1.5×10^5^ cells/ml.

Under a sterile hood we serially diluted 44 µl of each serum sample with PBS from 1:2 to 1:256 in a 96-well plate and added 10 µl of bacterial working solution to each dilution. We mixed 10 µl of bacterial working solution with 44 µl of PBS as positive control and used PBS only as negative control. The plate was covered and put on a plate shaker for 1 min at 150 rpm. It was incubated for 30 min at 37°C and again gently mixed on a plate shaker for 1 min at 150 rpm. Then, 250 µl of TSB, pre-warmed to 37°C, were added to all wells and the plate was again put on a plate shaker for 1 min at 100 rpm. Samples were measured in duplicates at 300 nm using a microplate reader (Biotek; µQuant Microplate Spectrophotometer, Winooski, USA) to determine the background absorbance at the starting point of bacterial growth. After an incubation of 12 h at 37°C the absorbance of the samples was measured again.

The absorbance readings of the negative controls at 0 h and 12 h of incubation should be similar. However, we noticed a slight discolouring in TSB during incubation when TSB was prepared 2 days or less before use, resulting in a drop of absorbance in negative controls by up to 14%. If the samples, the reagents or the plates were contaminated, a raise in absorbance would be expected. We therefore suggest using only TSB that has been prepared at least 2 days prior to the assay. To account for the affected plates, we subtracted the mean absorbance of negative controls at 0 h of incubation from the one at 12 h of incubation (=δC). We then subtracted absorbance of samples at 0 h of incubation from the ones at 12 h of incubation (=δA_i_, δA_j_, …) and added δC to each δA. The mean of the duplicates (=(δA_i_+δA_j_)/2=øD_i,j_) was used to calculate the bacterial killing capacity as 1−øD_i,j_/øP×100, with øP being the mean absorbance of the positive controls.

We used serial serum dilutions from 1:2 to 1:256 to identify optimal working dilutions for each species (French and Neuman-Lee, 2012). At one of these dilutions, samples should kill on average approximately 50% of bacteria. We did not detect such a dilution for the carnivore species tested, except for black-backed jackals, because in preliminary tests killing capacity systematically dropped from ∼100% to 0% between two serial dilutions (Fig. 4B). The drop in bacterial killing capacity for cheetahs, the majority of our samples, mostly occurred between dilutions 1:64 and 1:128. We therefore investigated this phenomenon in more detail and diluted additional aliquots of 20 randomly selected cheetahs to 1:70, 1:80 and 1:90. Still, we could not identify an optimal dilution for cheetahs, because the bacterial killing capacity was for all dilutions in most cases either 100% or 0% (Fig. 4C). Thus, we concluded that the assay allows the bacteria to grow to saturation if the plasma is not able to kill 100% of bacteria. This means that there might be a fine-tuned threshold dilution for each individual at which it cannot kill the bacteria any more. This is similar to the findings of a recent study on BKA in spotted hyenas (Flies et al., 2015), but differs from other studies that identified optimal working dilutions for each species (French and Neuman-Lee, 2012; Liebl and Martin, 2009). Because we could not identify an optimal dilution for most of the species, we performed serial dilutions for all samples and all species and attributed a rank to the last dilution before the drop to 0% killing, i.e. dilution 1:2 corresponded to rank 1, dilution 1:4 to rank 2, etc. Because we measured all samples as duplicates, we sometimes measured at one particular dilution 100% bacterial killing for one aliquot and 0% for the other one, resulting in a mean value of 50% for this dilution. Such individuals were assigned the mean of the ranks of both dilutions with 100% and 50% bacterial killing respectively. If bacterial killing did not reach 100% even at dilution 1:2, the rank score was 0. Consequently, all black-backed jackals had ranks of 0.

### Statistical analyses

All statistical analyses were performed using the open-source software R version 3.0.2 (R-Core-Team, 2015). To test whether phylogeny is a good predictor for bacterial killing capacity, we calculated mean BKA values for each species at each dilution. We then calculated a Euclidean distance matrix for all possible pairs of species. We used a Mantel test to compare this distance matrix with a published phylogenetic distance matrix (Nyakatura and Bininda-Emonds, 2012). The R package ‘clinfun’ (https://cran.r-project.org/web/packages/clinfun/) was used for the Jonckheere–Terpstra test, the R package ‘Skillings.Mack’ (https://cran.r-project.org/web/packages/Skillings.Mack/) for the Skillings–Mack test and the packages ‘ape’ (Paradis et al., 2004), ‘picante’ (Kembel et al., 2010) and ‘geiger’ (Harmon et al., 2008) for examination of whether phylogenetic relationships of species are a good predictor of the bacterial killing capacity of the species. Fig. 4 was created with the R package ‘ggplot2’ (Wickham, 2009).
